# Establishment and validation of a risk prediction model for urinary tract infection in elderly patients with type 2 diabetes mellitus

**DOI:** 10.3389/fendo.2025.1557185

**Published:** 2025-07-23

**Authors:** Yaqiang Li, Lin Li, Lili He

**Affiliations:** ^1^ Department of Nosocomial Awareness, Lixin County Hospital of Traditional Chinese Medicine, Bozhou, China; ^2^ Department of Neurology, People’s Hospital of Lixin County, Bozhou, China

**Keywords:** urinary tract infection, type 2 diabetes mellitus, nomogram, decision curve analysis, diabetes mellitus

## Abstract

**Objectives:**

This study aimed to identify the risk factors for urinary tract infection (UTI) in elderly patients with type 2 diabetes mellitus (T2DM) and to develop and validate a nomogram that predicts the probability of UTI based on these factors.

**Methods:**

We collected clinical data from patients with diabetes who were aged 60 years or older. These patients were then divided into a modeling population (n=281) and an internal validation population (n=121) based on the principle of random assignment. LASSO regression analysis was conducted using the modeling population to identify the independent risk factors for UTI in elderly patients with T2DM. Logistics univariate and multifactor regressions were performed by the screened influencing factors, and then column line graph prediction models for UTI in elderly patients with T2DM were made by these influencing factors, using receiver operating characteristic curve and area under curve, C-index validation, and calibration curve to initially evaluate the model discrimination and calibration. Model validation was performed by the internal validation set, and the ROC curve, C-index and calibration curve were used to further evaluate the column line graph model performance. Finally, using DCA (decision curve analysis), we observed whether the model could be used better in clinical settings.

**Results:**

The study enrolled a total of 402 patients with T2DM, of which 281 were in the training cohort, and 70 of these patients had UTI. Six key predictors of UTI were identified: “HbA1c ≥ 6.5%” (OR, 1.929; 95%CI, 1.565-3.119; P =0.045), “Age ≥ 65y” (OR, 3.170; 95% CI, 1.507-6.930; P=0.003), “DOD ≥ 10y” (OR, 2.533; 95% CI, 1.727-3.237; *P* = 0.036), “FPG” (OR, 2.527; 95% CI, 1.944-3.442; *P* = 0.000), “IUC” (OR, 2.633; 95%CI, 1.123-6.289; *P* = 0.027), and “COD” (OR, 1.949; 95%CI, 1.623-3.889; *P* = 0.041). The nomogram demonstrated a high predictive capability with a C-index of 0.855 (95% CI, 0.657-0.976) in the development set and 0.825 (95% CI, 0.568-0.976) in the validation set.

**Conclusions:**

Our nomogram, incorporating factors such as “HbA1c ≥ 6.5%,” “Age ≥ 65y”, “FPG”, “DOD ≥ 10y”, “COD”, and “IUC”, provides a valuable tool for predicting UTI in elderly patients with T2DM. It offers the potential for enhanced early clinical decision-making and proactive prevention and treatment, reflecting a shift towards more personalized patient care.

## 1 Introduction

Diabetes mellitus (DM) is a widespread condition that involves abnormalities in the metabolism of glucose and insulin (1). Among these, type 2 diabetes mellitus (T2DM) is the most prevalent, primarily characterized by high blood sugar levels due to the body’s insufficient insulin production or ineffective use of insulin (2, 3). Diabetes is a widespread chronic condition that poses an increasing concern for patients and healthcare professionals across the globe. Recent epidemiological surveys indicate that globally, approximately 828 million adults aged 18 years and older are expected to have diabetes in 2022, marking an increase of 630 million since 1990 (4). The economic burden on public health due to diabetes is on the rise, primarily attributed to the complications associated with the disease. Individuals with diabetes face an elevated risk of microvascular complications, including nephropathy, neuropathy, urinary tract infection (UTI), and retinopathy (5). DM has increasingly been recognized as a growing public health issue on a global scale. In the year 2019, the prevalence of DM worldwide was approximated at 9.3%, affecting around 463 million individuals. Projections indicate a rise to 10.2% (578 million individuals) by the year 2030, and further escalation to 10.9% (700 million individuals) by 2045 (6, 7). Furthermore, the population of elderly individuals aged 60 years and older who are diagnosed with diabetes is experiencing a swift rise, primarily attributed to the aging demographic (8). This group has emerged as the most significantly impacted by diabetes, with type 2 DM representing more than 90% of cases (9).

UTI frequently affect elderly patients with diabetes, with research showing that these infections occur 1.5 to 4 times more often in them than in those without diabetes (10, 11). The clinical manifestations of UTI in patients primarily include urinary pain, urgency, and frequency. However, if pyelonephritis is left untreated or inadequately treated, especially in individuals with DM and additional complicating factors, it can result in severe complications. Therefore, the early identification of UTI risk in elderly patients with DM holds significant clinical importance. Research demonstrates that individuals aged 65 and older are at an increased risk of UTI (12). UTI are frequently observed in elderly individuals with diabetes. Previous studies indicate that approximately 43% of patients with type 2 DM aged 60 years or older show the presence of bacteria in their urine (13, 14). A comprehensive systematic review and meta-analysis indicated that the prevalence of UTI among individuals diagnosed with type 2 DM is 11.5%. Moreover, the incidence rate was found to increase with advancing age (15). Older adults may experience diabetes and UTI that develop slowly (16). This gradual onset can make these conditions difficult to detect. If UTI are not treated promptly, they may progress to serious complications such as pyelonephritis and cystitis (17). These complications can not only extend the patient’s hospital stay and escalate treatment costs, but they may also adversely impact the patient’s quality of life and overall prognosis (18, 19). Therefore, it is essential to develop appropriate management strategies aimed at preventing UTI in elderly patients with diabetes.

Current studies reported in the literature on UTI in patients with DM primarily focus on traditional risk factors, such as age, gender, body mass index, and glycemic control (15, 20, 21). However, most of these studies are limited to examining the independent risk factors associated with the occurrence of UTI in patients with DM, which hampers accurate predictions of the potential risks leading to UTI in this population. Each patient should be individually assessed in clinical practice by considering multiple parameters to predict the risk of developing a UTI after DM. However, this integration of parameters is currently missing in clinical practice. A nomogram serves as a graphical statistical instrument designed to evaluate and compute the likelihood of specific clinical outcomes for patients through the application of a continuous scoring system. In recent years, this tool has gained traction as a predictive methodology particularly in the context of DM (22, 23). Therefore, the current study aims to identify the independent predictors associated with UTI in patients with DM and to establish and validate a nomogram that integrates clinical data and laboratory variables for predicting the likelihood of developing UTI in this patient population.

## 2 Materials and methods

### 2.1 Study design and subjects

**Figure 1:**
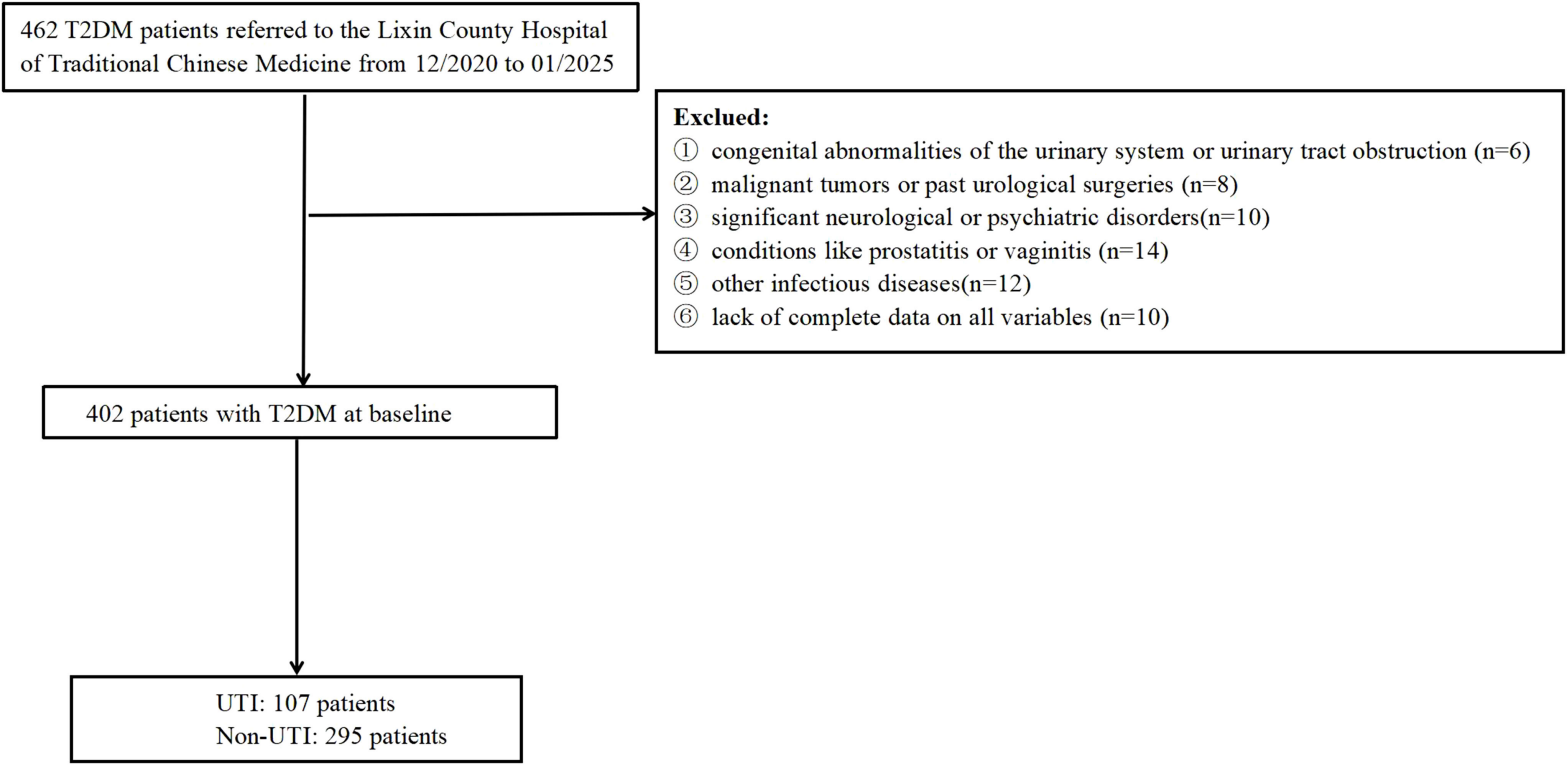
Flowchart of participant selection. T2DM, type 2 diabetes mellitus; UTI, urinary tract infection.

This retrospective cohort study was conducted in accordance with the principles outlined in the Declaration of Helsinki and obtained approval from the Ethics Committee at the Lixin County Hospital of Traditional Chinese Medicine. Due to the study’s retrospective nature, the review board waived the requirement for written informed consent. In this study, we systematically enrolled individuals diagnosed with Diabetes Mellitus from December 2020 to January 2025 at the Lixin County Hospital of Traditional Chinese Medicine. The criteria for inclusion are outlined as follows: (1) Participants must be aged 60 years or above; (2) Diagnosis of Type 2 DM must align with the standards specified by the World Health Organization (24); (3) Individuals should exhibit full awareness and possess no impediments to effective communication. The exclusion criteria include: (1) congenital abnormalities of the urinary system or urinary tract obstruction; (2) malignant tumors or past urological surgeries; (3) significant neurological or psychiatric disorders; (4) major organ dysfunction; (5) conditions like prostatitis or vaginitis; (6)other infectious diseases. The identification of UTI was established during the hospital stay following surgical intervention when patients exhibited isolated acute dysuria, or presented with fever accompanied by at least one of the subsequent symptoms: increased urinary frequency, urgency, or retention; incontinence; visible hematuria; alterations in urine properties; or tenderness in the suprapubic region or costovertebral angle. This diagnosis was further supported by the detection of ≥10^3 colony-forming units of any organism in the urine culture (25, 26). All enrolled patients were divided into the training cohort and validation cohort based on the pre-seeded random number (123) generator in R software(version 4.4.3). Finally, the patients were randomly divided into training (n=281) and validation (n=121) cohorts based on the ratio of 7:3. At last, the study included 402 cases with DM in total (**Figure 1**).

### 2.2 Clinical data collection

Baseline characteristics include demographic data (including age, gender, education years, and body mass index), clinical data [including the history of hypertension, duration of diabetes (DOD), smoking, and drinking] were collected for patients at admission. Venous blood was drawn from patients the next morning after admission. We measured triglycerides(TG), triglyceride(TC), high-density lipoprotein (HDL), low-density lipoprotein (LDL), apolipoprotein A (ApoA), and apolipoprotein B (ApoB), glycated hemoglobin A1c (HbA1c), serum creatinine (SCr), fasting blood glucose (FPG), and blood urea nitrogen (BUN). In addition, data were also collected on indwelling urinary catheters (IUC) and various complications related to diabetes, such as diabetic nephropathy, peripheral vascular disease, retinopathy, and diabetic foot ulcers.

### 2.3 Statistical analysis

Statistical analyses were conducted utilizing the R statistical software (version 4.4.3; R Foundation for Statistical Computing, Vienna, Austria). Continuous variables were summarized using the mean ± standard deviation (SD) or the median with the interquartile range (IQR). In contrast, categorical variables were represented as frequencies with corresponding percentages. To evaluate the differences in baseline characteristics across the groups, independent sample t-tests were utilized for continuous variables that met parametric assumptions, while the Mann-Whitney U tests were employed for non-parametric continuous variables. For categorical variables, the chi-squared test or Fisher’s exact test was applied, depending on the suitability of the data. LASSO (Least absolute shrinkage and selection operator) regression analysis was first performed using the (glmnet) package of R language software (version 4.4.3) to screen out statistically significant variables, i.e., age>65y, females, DOD>10y, FPG, IUC, and complications of diabetes (COD) were influential risk factors for UTI in the elderly with DM. Subsequently, multivariate analyses were conducted to determine the independent predictors of UTI in elderly patients diagnosed with diabetes. The independent variables identified through binary logistic regression analysis were entered into R software, where the nomogram prediction model was developed utilizing the “rms” package. The model’s discriminatory capability was assessed by calculating the area under the receiver operating characteristic (ROC) curve. In order to assess the accuracy of the model, a bootstrapping technique involving 1,000 resamples was utilized, which facilitated the creation of a calibration curve. The practical applicability and actual advantages of the model were evaluated through decision curve analysis (DCA). A p-value of less than 0.05 was deemed to indicate statistical significance.

## 3 Results

The study enrolled patients with T2DM from participating centers between December 2020 to January 2025. Initially, we recruited 462 participants. However, we subsequently excluded 60 participants. This included 10 individuals who did not provide complete data on all variables, as well as 50 participants who met the exclusion criteria. These criteria included congenital abnormalities of the urinary system, urinary tract obstruction, malignant tumors, past urological surgeries, significant neurological or psychiatric disorders, conditions such as prostatitis or vaginitis, and various other infectious diseases. All participants were randomly divided into a training cohort (n=281) and a validation cohort (n=121) using the R programming language, maintaining a ratio of 7:3. **Table 1** presents the characteristics of the training cohort and the validation cohort, showing similar attributes. Among the 402 elderly individuals, 107(26.62%) exhibited UTI, with 70(24.91%) in the training cohort and 37 (30.58%) in the validation cohort. **Table 1** displays the characteristics of both the training and validation cohorts, which are similar.

**Table 1:** Demographics and clinical characteristics of the training and validation cohort.

	All patients (n=402)	Validation cohort (n=121)	Training cohort (n=281)	*P*-value
Demographic characteristics				
Females, n (%)	231 (57.46)	70 (57.85)	161 (57.29)	0.918
Education years, median (IQR)	5 (3,7)	5 (3,7)	5 (3,7)	0.637
Age≥65y, n (%)	205 (50.99)	67 (55.37)	138 (49.11)	0.297
BMI>24.0%, n (%)	232 (57.71)	75 (61.98)	157 (55.87)	0.304
Vascular risk factors (%)				
Hypertension, n (%)	270 (67.16)	79 (65.29)	191 (67.97)	0.682
Current smoking, n (%)	126 (31.34)	37 (30.58)	89 (31.67)	0.921
DOD>10y, n (%)	190 (47.26)	61 (50.41)	129 (45.91)	0.471
Alcohol consumption, n (%)	138 (34.33)	36 (29.75)	102 (36.30)	0.249
Laboratory parameters (IQR)				
TG>1.7 mmol/L, n (%)	157 (39.05)	42 (34.71)	115 (40.93)	0.289
TC>6.0 mmol/L, n (%)	159 (39.55)	49 (40.49)	110 (39.15)	0.887
HDL, mmol/L, median (IQR)	1.03 (0.86,1.25)	1.02 (0.87,1.23)	1.03 (0.86,1.26)	0.727
LDL, mmol/L, median (IQR)	2.54 (1.98,3.18)	2.51 (1.98,3.08)	2.57 (1.99,3.2)	0.462
APA, g/L, median (IQR)	1.27 (1.1,1.45)	1.27 (1.11,1.44)	1.27 (1.09,1.46)	0.899
APB, g/L, median (IQR)	0.86 (0.69,1.01)	0.85 (0.67,1.01)	0.86 (0.7,1.01)	0.232
HbA1c>6.5%, n (%)	133 (33.08)	48 (39.67)	85 (30.25)	0.084
BUN, mmol/L, median (IQR)	7.17 ± 2.17	7.07 ± 1.96	7.21 ± 2.26	0.54
SCr, μmmol/L, median (IQR)	107.79 ± 31.83	105.44 ± 31.32	108.8 ± 32.05	0.328
FPG, mmol/L, median (IQR)	8.13 (7.54,9.03)	8.4 (7.72,9.05)	8.05 (7.49,8.98)	0.046
IUC, n (%)	163 (40.55)	52 (42.98)	111 (39.50)	0.589
COD, n (%)	148 (36.82)	47 (38.84)	101 (35.94)	0.66

TC, triglyceride; TG, triglycerides; HDL, high-density lipoprotein; LDL, low-density lipoprotein; ApoA, apolipoprotein A; ApoB, apolipoprotein B; HbA1c, glycated hemoglobin A1c; IUC, indwelling urinary catheter; SCr, serum creatinine;FPG, fasting blood glucose; BMI, body mass index; BUN, blood urea nitrogen; DOD, duration of diabetes.

**Figure 2:**
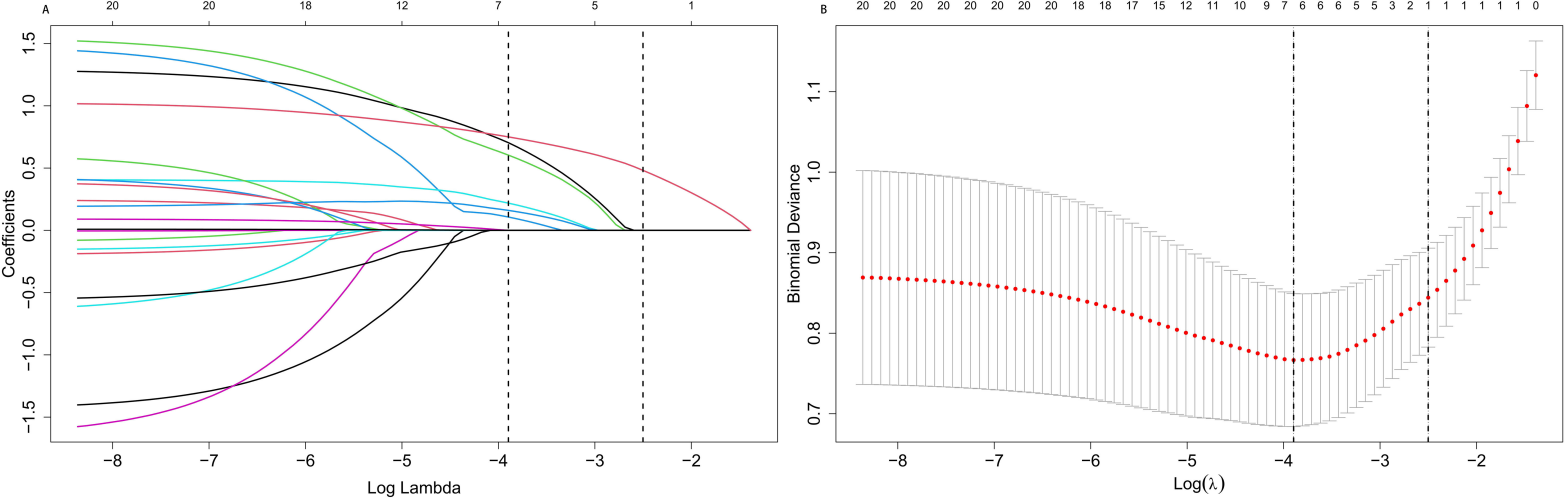
Screening variables based on LASSO regression. **(A)** All relevant parameters were included in the LASSO analysis, resulting in the plotting of their corresponding coefficient curves. **(B)** The coefficient profile distribution of the log(λ) sequence derived from LASSO regression was plotted against log(λ) to illustrate the C index. The 1 standard error of the minimum criterion (1-SE criterion) and the minimum criterion indicate the optimal value, with the lambda of the minimum criterion resulting in five features with non-zero coefficients.

**Figure 3:**
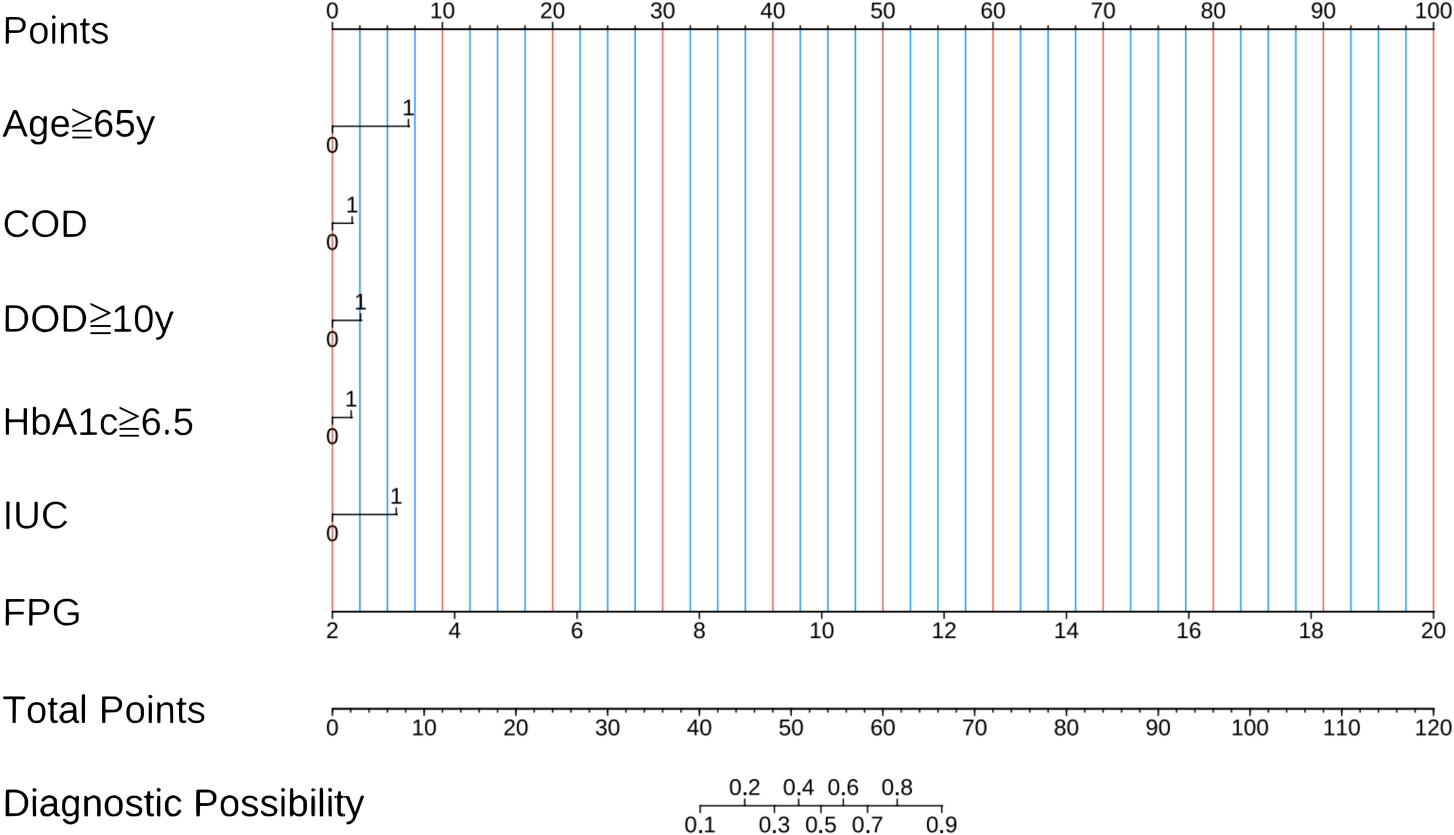
Nomogram for predicting UTI in elderly patients with diabetes. UTI, urinary tract infection; HbA1c, glycated hemoglobin A1c; IUC, indwelling urinary catheter; FPG, fasting blood glucose; DOD, duration of diabetes.

From the 20 features analyzed, we identified 6 potential predictors based on a training set of 281 patients, resulting in a ratio of 3.3:1 (**Figures 2A, B**). These predictors were associated with non-zero coefficients in the LASSO logistic regression model. A predictive model was constructed using five predictors identified from the original nineteen by LASSO logistic regression, which ensures concise and reliable forecasting in our study. A nomogram was created to visually represent the predictive model. It helps clinicians easily calculate outcome probabilities by linking each variable’s value to its points (**Figure 3**). In the multivariate analysis of the training set (**Table 2**), six potential predictors were identified through the LASSO logistic regression model. Specifically, “HbA1c≥6.5%” (OR, 1.929; 95%CI, 1.565-3.119; P =0.045), “Age≥65y” (OR, 3.170; 95% CI, 1.507-6.930; P=0.003), “DOD≥10y” (OR, 2.533; 95% CI, 1.727-3.237; p =0.036), “FPG” (OR, 2.527; 95% CI, 1.944-3.442; P=0.000), “IUC” (OR, 2.633; 95%CI, 1.123-6.289;P=0.027), and “COD” (OR, 1.949; 95%CI, 1.623-3.889;P=0.041) were evaluated. Notably, “HbA1c≥6.5%,” “Age≥65y”, “FPG”, “DOD≥10y”, “COD”, and “IUC” were found to be statistically significant predictors, with p-values below the conventional 0.05 threshold.

**Table 2:** Logistics regression univariate and multifactor analysis.

Variables	Univariate logistic regression analysis		Multivariate logistic regression analysis	
	OR (95% CI)	*P* value	OR(95% CI)	*P* value
Demographic characteristics				
Females, n (%)	1.112(0.647-1.924)	0.702		
Education years, median (IQR)	1.003(0.935-1.058)	0.924		
Age≥65y, n (%)	3.199(1.813-5.805)	0.000	3.170 (1.507-6.930)	0.003
BMI>24.0%, n (%)	1.355(0.784-2.373)	0.281		
Vascular risk factors (%)				
Hypertension, n (%)	0.872(0.495-1.563)	0.641		
Current smoking, n (%)	1.015(0.572-1.84)	0.96		
DOD≥10y, n (%)	3.215(1.835-5.765)	0.000	2.533 (1.727-3.237)	0.036
Alcohol consumption, n (%)	1.034(0.592-1.835)	0.907		
Laboratory parameters (IQR)				
TG>1.7 mmol/L, n (%)	1.638(0.949-2.83)	0.076		
TC>6.0 mmol/L, n (%)	1.678(0.97-2.902)	0.064		
HDL, mmol/L, median (IQR)	1.048(0.41-2.58)	0.92		
LDL, mmol/L, median (IQR)	1.131(0.824-1.547)	0.442		
APA, g/L, median (IQR)	1.103(0.405-2.95)	0.846		
APB, g/L, median (IQR)	1.049(0.513-1.862)	0.872		
HbA1c>6.5%, n (%)	2.51(1.427-4.419)	0.001	1.929 (1.565-3.119)	0.045
BUN, mmol/L, median (IQR)	1.034(0.916-1.164)	0.587		
SCr, μmmol/L, median (IQR)	1.002(0.994-1.011)	0.64		
FPG, mmol/L, median (IQR)	2.674(2.083-3.585)	0	2.527 (1.944-3.442)	0.000
IUC, n (%)	2.429(1.404-4.237)	0.002	2.633 (1.123-6.289)	0.027
COD, n (%)	2.575(1.483-4.5)	0.001	1.949 (1.623-3.889)	0.041

TC, triglyceride; TG, triglycerides; HDL, high-density lipoprotein; LDL, low-density lipoprotein; ApoA, apolipoprotein A; ApoB, apolipoprotein B; HbA1c, glycated hemoglobin A1c; IUC, indwelling urinary catheter; SCr, serum creatinine;FPG, fasting blood glucose; BMI, body mass index; BUN, blood urea nitrogen; DOD, duration of diabetes.

**Figure 4:**
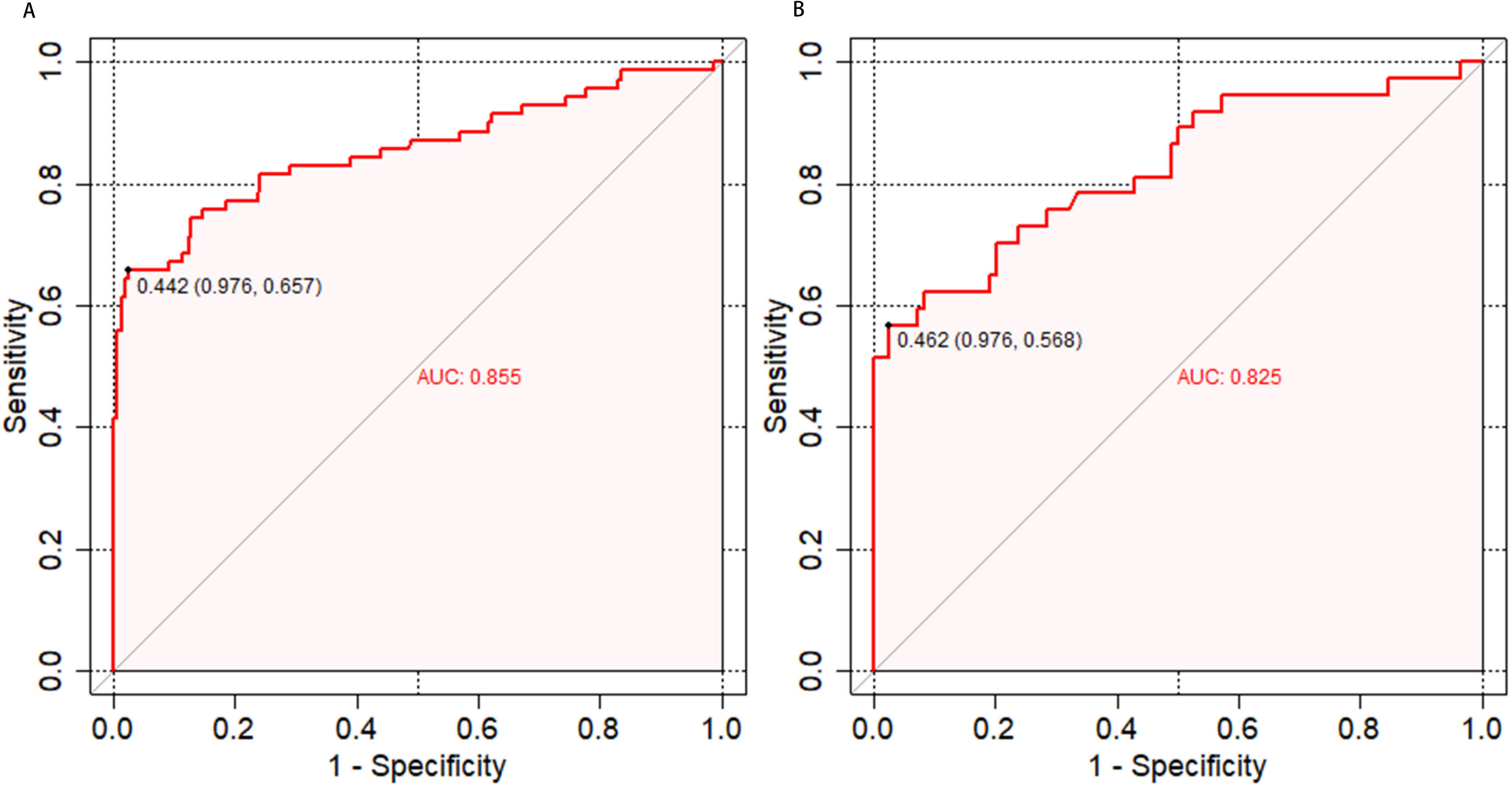
Receiver operating characteristic curves for risk of UTI in elderly patients with diabetes. **(A)** shows the ROC Curve for the Training set. **(B)** depicts the ROC Curve for the Validation set. Sensitivity and specificity of several risk thresholds of the prediction model are plotted. UTI, urinary tract infection; ROC, receiver operating characteristic.

**Figure 5:**
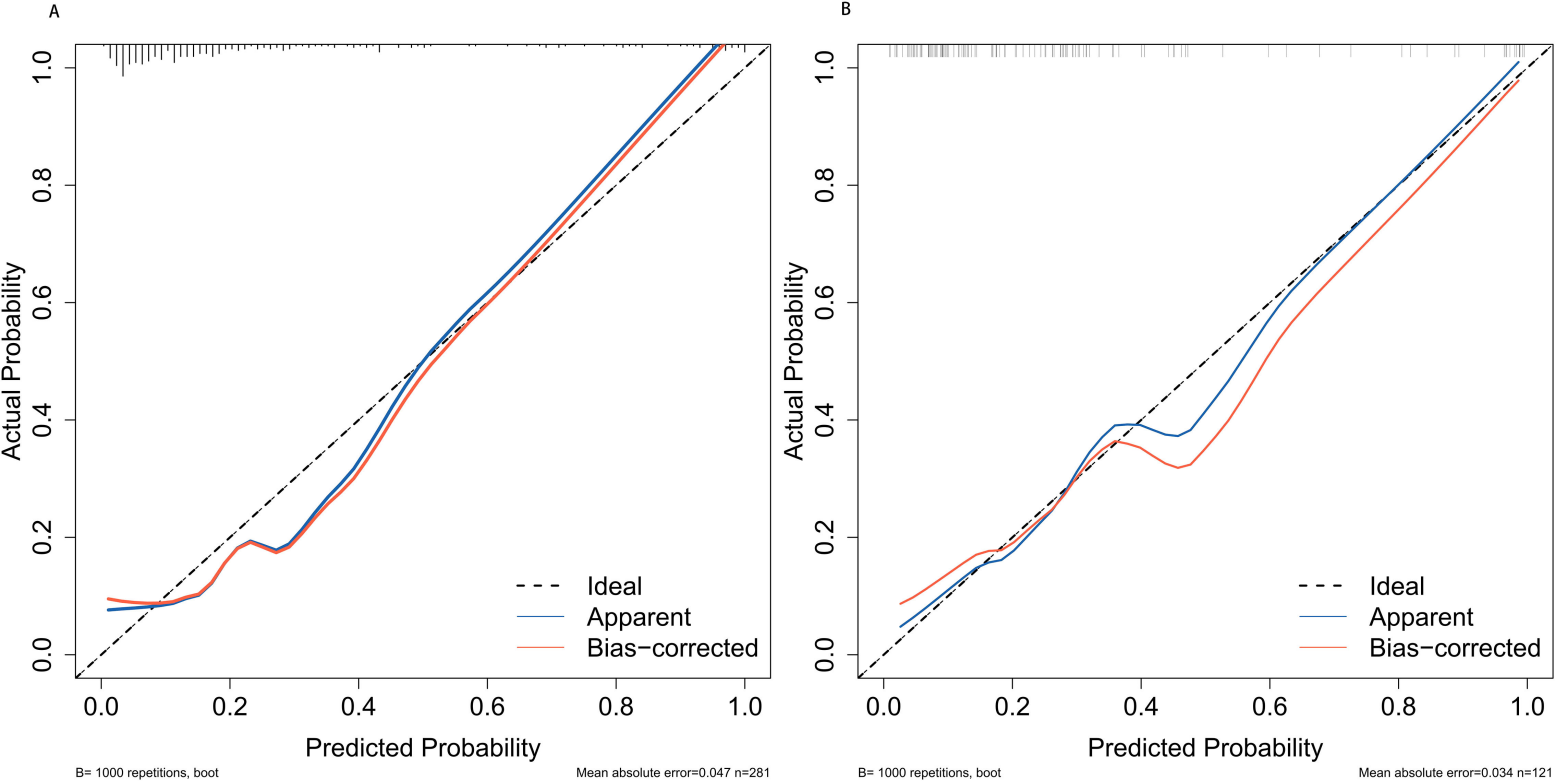
Calibration plots for predicting postoperative UTI in elderly patients with diabetes for both the training cohort **(A)** and the validation cohort **(B)**. UTI, urinary tract infection.

**Figure 6:**
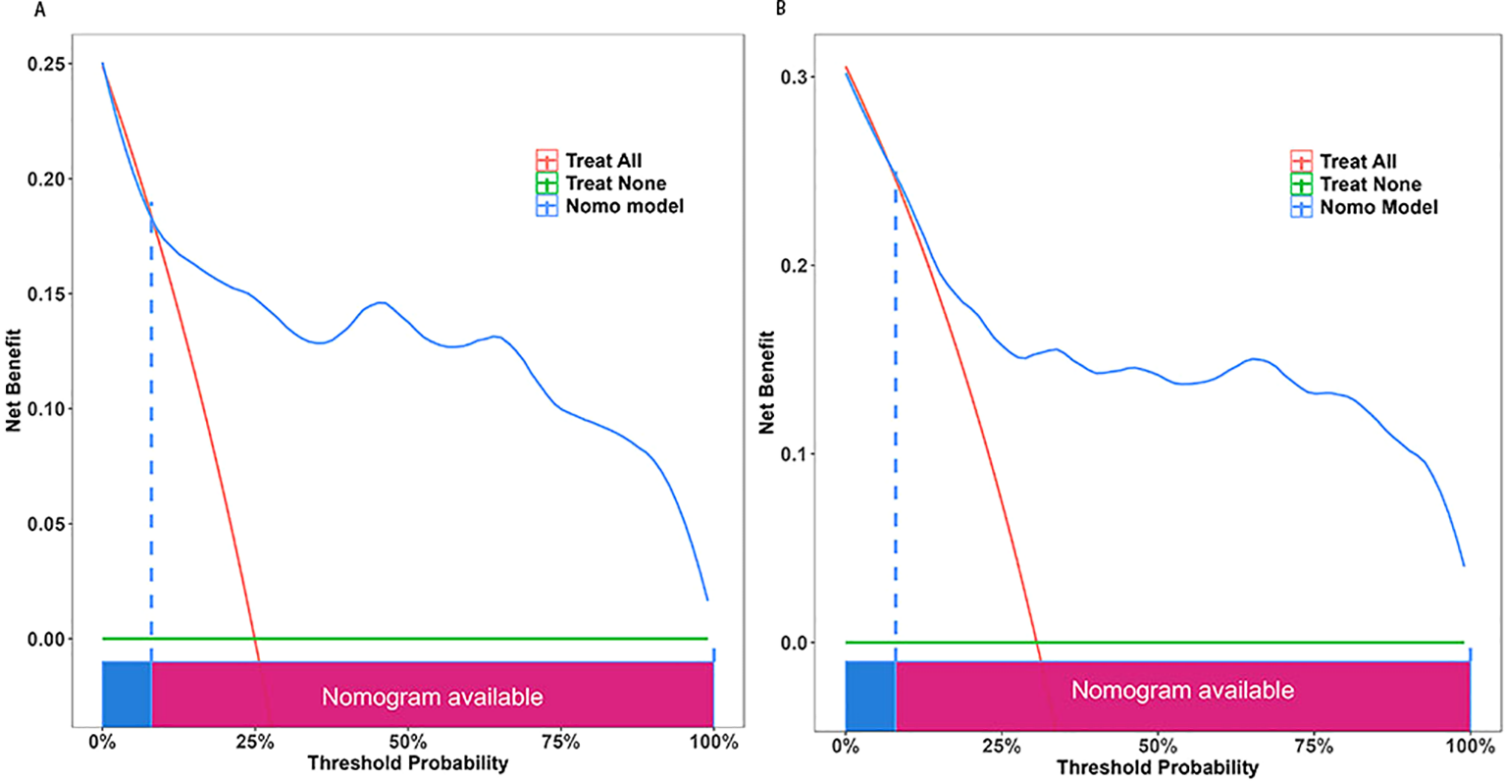
The Decision Curve Analysis (DCA) was performed on the nomogram designed to forecast UTI following T2MD within both the training cohort **(A)** and the validation cohort **(B)**. The x-axis illustrates the probability threshold. The y-axis indicates the net benefit. The green line indicates that all patients tested negative and did not receive any treatment, resulting in a net benefit of zero. The red line indicates that every patient undergoing surgical intervention is expected to experience a UTI. The blue line represents the overall advantage provided by the nomogram. UTI, urinary tract infection; T2MD.

The discrimination of the nomogram, evaluated on the training set, was quantified by calculating the c-index, which was found to be 0.855 (95% CI, 0.657-0.976), indicating strong predictive capability (**Figure 4A**). Similarly, the assessment of the validation set yielded a c-index of 0.825 (95% CI, 0.568-0.976), demonstrating the nomogram’s consistent performance across different datasets (**Figure 4B**). **Figure 5** presents a calibration plot that compares the mortality predictions generated by the nomogram with actual observations from the same training set. The calibration plot indicates a strong predictive accuracy of the nomogram (**Figure 5A**). Furthermore, the model was validated externally with the test cohort, resulting in a c-index of 0.825 (95% CI, 0.568-0.976). Considering that a c-index exceeding 0.75 is typically regarded as indicative of reliable discrimination, this nomogram demonstrated commendable performance in both the training and validation sets. The favorable calibration of the nomogram was further confirmed by the validation set (**Figure 5B**). As illustrated in **Figure 6**, the decision curve analysis revealed that within the training set, threshold probabilities from 8.0% to 100% (**Figure 6A**), and within the validation set, from 8.5% to 100% (**Figure 6B**), the application of the nomogram for forecasting Postoperative UTI yielded a superior net benefit compared to the “treat all” or “treat none” approaches. This finding underscores the clinical relevance of utilizing the nomogram.

## 4 Discussion

This study presents novel, accessible, and highly accurate columnar line graphs designed to predict the risk of developing UTI in elderly individuals with diabetes. To the best of our knowledge, there has been no previous research focused on the development and examination of a columnar line graph prediction model specifically aimed at forecasting the occurrence of UTI in this demographic. The columnar line plots exhibited notable consistency in both the simulated and validated groups, underscoring their significant clinical implications. It has the potential to inform and influence decisions made in a clinical setting (27). Column line graphs effectively meet the need for integrated biological and clinical models, as well as the advancement of personalized medicine, which aims to provide customized predictions for individual patients (28).

DM is a chronic condition characterized by sustained high levels of blood glucose, which fosters an environment conducive to bacterial growth and reproduction (29). Prolonged hyperglycemia induces damage to the autonomic nerves, which subsequently contributes to the development of a neurogenic bladder. The accumulation of urine within the bladder creates an environment conducive to bacterial proliferation, thereby increasing the risk of urinary tract infections (30). UTI represent the most frequently encountered bacterial illness among the elderly demographic. With advancing age, older individuals with diabetes undergo a notable deterioration in their physiological and immune capabilities, resulting in a substantial reduction in their ability to combat infectious agents (31). Additionally, in older adults, the mucosal epithelium of both the urinary and reproductive systems undergoes degeneration, which subsequently heightens the susceptibility to UTI (32). The presence of infection hinders the regulation of blood glucose levels, thereby creating a detrimental cycle that exacerbates the overall health of individuals with diabetes. This situation leads to persistent health issues and a range of complications, including diabetic nephropathy, cerebrovascular diseases, diabetic retinopathy, diabetic macroangiopathy, and diabetic neuropathy (13). These complications worsen the clinical management of older adults diagnosed with type 2 DM and have the potential to increase the indirect mortality rates among these patients (32).

In the present study, a comprehensive nomogram was created employing variables such as females, age≥65y, PFG, ICU, DOD>10y, and complications associated with DM. This model seeks to proficiently predict the incidence of UTI in patients afflicted with DM. The remarkable capacity of the nomogram to differentiate and calibrate was distinctly illustrated in the training cohort and subsequently validated through external evaluations. Ultimately, the DCA, which functions as a specialized tool for evaluating the clinical value of a nomogram, suggested that our nomogram possesses considerable promise for predicting UTI in clinical settings. A significant advantage of our model is its foundation on pre-treatment parameters, all of which can be acquired prior to the commencement of any therapeutic measures. This not only enhances its clinical relevance but also offers healthcare practitioners, patients, and their families a pragmatic framework, facilitating a well-informed decision-making process from the outset.

Both univariate and multivariate analyses indicated that age (≥65y), PFG, ICU, DOD(≥10y), and complications of DM correlated with a higher incidence of UTI in this investigation. It is possible that age≥65years is a risk factor for UTI in elderly patients with diabetes, probably because with age, patients have a higher probability of being associated with underlying diseases, and their body functions and immune functions decline significantly, resulting in a significantly reduced ability to fight pathogenic bacteria, thus increasing the risk of UTI. The correlation between age and the risk of DM infections has been confirmed by previous studies (33, 34). DOD and FPG as risk factors for UTI in patients with diabetes may be due to the fact that as the duration of diabetes increases, the fluctuation of blood glucose levels is more obvious and the effect of blood glucose control is not satisfactory. While FPG reflects the level of blood glucose control in patients, the longer the duration of diabetic disease and the higher the FPG, the greater the risk of the patient being in a state of hyperglycemia. Excessive glucose in the blood lowers immune function, and elevated sugar levels in the blood and urine are more conducive to bacterial growth, thus increasing the risk of UTI (35). A study by Aswani et al. found that the risk of urinary tract infections nearly doubles, increasing by 1.9 times, for every 10 years of diabetes (36). In addition, diabetic nephropathy, peripheral vasculopathy, retinopathy, diabetic foot, and other complications are common in elderly patients with diabetes, which are also effective indicators reflecting the effectiveness of patients’ blood glucose control and the severity of their conditions. Patients with the above-mentioned complications tend to have more severe conditions, higher blood glucose levels, a decline in immune function, and a significantly higher risk of UTI (37). Patients with IUC are required to undergo invasive procedures involving these devices. The direct connection between the indwelling catheter and the external environment heightens the risk of pathogenic bacterial invasion. Bacteria are prone to colonize the biofilm that forms on the catheter’s surface, which subsequently increases the likelihood of invasive infections in the urethra, thereby elevating the risk of UTI (38, 39).

Clinical practice strengthens daily dietary guidance for older patients and provides timely nursing care to ensure they consume sufficient nutrition. It is crucial to provide special attention to patients with diabetes with a long illness duration. This includes enhancing their health awareness, improving the quality of care, and actively reducing external factors that could lead to complications of UTI. For patients requiring an indwelling catheter, the necessity of its use can be clarified through multidisciplinary consultation. The principle of avoiding indwelling catheters should be implemented whenever possible to effectively reduce the urinary catheter indwelling rate and prevent inappropriate use. Additionally, an early extraction reminder system can be established to promote timely catheter removal and minimize the duration of catheter indwelling. Aggressively monitor and promptly control blood glucose in patients with high FPG levels and implement early preventive measures to reduce the incidence of urinary tract infections in patients with T2DM (40).

Our study presents several limitations that merit attention. Firstly, it is important to note that this investigation was conducted as a small-sample, single-center retrospective cohort study. To enhance the statistical robustness of our findings, we selected only those variables that exhibited P values less than 0.05 in univariate analyses as potential candidates for multivariate regression analyses. Secondly, the nomogram has not undergone validation within an external cohort. Consequently, it is imperative to conduct a multicenter prospective study to assess the nomogram’s applicability prior to its implementation in clinical settings. Finally, despite our efforts to account for numerous variables in the formulation of our predictive model, we cannot dismiss the possibility of certain unmeasured baseline variables, which might impact the occurrence of UTI following DM.

## 5 Conclusions

In summary, the developed nomogram aims to predict the likelihood of UTI in elderly patients with T2DM. It uses several variables, including “HbA1c ≥ 6.5%,” “Age ≥ 65 years,” “FPG,” “DOD ≥ 10 years,” “COD,” and IUC. The model relies on available pre-treatment indicators and aims to provide practical guidance for early decision-making in clinical contexts. However, it is essential to apply it cautiously and further validate its utility across various patient populations.

## Data Availability

The original contributions presented in the study are included in the article/supplementary material. Further inquiries can be directed to the corresponding author.
